# Attitude toward livestock farming does not influence the earlier observed association between proximity to goat farms and self-reported pneumonia

**DOI:** 10.1097/EE9.0000000000000041

**Published:** 2019-04-12

**Authors:** Floor Borlée, C. Joris Yzermans, Floor S. M. Oostwegel, François Schellevis, Dick Heederik, Lidwien A. M. Smit

**Affiliations:** a Institute for Risk Assessment Sciences, Utrecht University, Utrecht, The Netherlands; b Netherlands Institute for Health Services Research, NIVEL, Utrecht, The Netherlands; c Department of General Practice & Elderly Care Medicine, Amsterdam Public Health Research Institute, VU University Medical Center, Amsterdam, The Netherlands.

## Abstract

Attitudes toward environmental risks may be a source of bias in environmental health studies because concerns about environmental hazards may influence self-reported outcomes.

**Objective:**

The main aim was to assess whether earlier observed associations between proximity to goat farms and self-reported pneumonia were biased by participants’ attitude toward farming.

**Methods:**

We developed an attitude-score for 2,457 participants of the Dutch Livestock Farming and Neighbouring Residents’ Health Study (veehouderij en gezondheid omwonenden) by factor analysis of 13 questionnaire items related to attitude toward livestock farming. Linear regression analysis was used to assess associations between attitude and potential determinants. The effect of attitude on the association between goat farm proximity and pneumonia was analyzed by evaluating (1) misclassification of the outcome, (2) effect modification by attitude, and (3) exclusion of participants reporting health problems due to farms in their environment.

**Results:**

In general, the study population had a positive attitude toward farming, especially if participants were more familiar with farming. Older participants, females, ex-smokers, and higher-educated individuals had a more negative attitude. Both self-reported respiratory symptoms and exposure to livestock farms were associated with a more negative attitude. Misclassification of self-reported pneumonia was nondifferential with regard to participants’ attitude. Furthermore, no indication was found that the association between proximity to goat farms and pneumonia was modified by attitude. Excluding subjects who attributed their health symptoms to livestock farms did also not change the association.

**Conclusions:**

The association between goat farm proximity and pneumonia was not substantially biased by study participants’ attitude toward livestock farming.

## Abstract 2

Supplemental Digital Content is available in the text.

## Introduction

Environmental hazards—such as air or drinking water pollution—may be a source of concern in exposed individuals.^1,2^ In epidemiologic studies, information on health outcomes is often self-reported, which has well-documented limitations such as recall bias and social desirability bias.^3^ Study participants’ attitudes toward environmental risks may be a source of information bias as well because concerns about environmental hazards may influence self-reported outcomes. Moffatt et al^1^ describes such “awareness bias” as the propensity to report more illness and symptoms as a result of proximity to a potential hazard, in the absence of a biologic effect. Perception of exposure, causal beliefs and concerns, and media coverage play an important role in symptom reporting.^4–8^

Actual or perceived exposure to a hazard, and cultural and social factors may influence someone’s risk perception, which results in a variation of attitudes toward a potential environmental risk among individuals.^9^ Marcon et al^2^ found that determinants of environmental risk perception mainly comprise demographic, socioeconomic, and exposure indicators. However, the authors did not investigate whether risk perception affected epidemiologic associations between environmental pollution and self-reported health outcomes.^2^

There is an ongoing debate about intensive livestock farming and potential health risks for surrounding populations.^10–14^ The Netherlands is a small country with one of the highest population densities in the world in combination with one of the highest livestock densities.^15^ A small survey (n = 1,090) on the public’s view on intensive livestock farming showed disagreement among the Dutch general population about large-scale intensive farming.^16^ Most arguments against intensive livestock farming were focused on animal welfare, and potential risks for public health.

The veehouderij en gezondheid omwonenden (VGO) study (Dutch acronym for Livestock Farming and Neighbouring Residents’ Health) investigated a wide range of health risks (respiratory health, zoonotic infections, and antimicrobial resistance) among residents living in close proximity of livestock farms in the Netherlands.^17–24^ One of the main findings was a higher risk of pneumonia for residents living in close proximity to goat farms.^22,24^ Pneumonia was defined based on questionnaire data^22^ and/or a diagnosis of pneumonia by the general practitioner (GP), recorded in the Electronic Medical Record (EMR).^22,24^ As a direct policy implication, five Dutch provinces have stopped issuing building permits for goat farms, awaiting further evidence. However, one can raise the criticism that potential awareness bias—overreporting of pneumonia by exposed individuals—may have resulted in a biased association.

In the present analysis, we constructed an “attitude toward farming” score as a proxy for awareness of farming as an environmental hazard. The main aim of the current study is to assess whether the earlier observed association^22^ between proximity to goat farms and pneumonia was biased by participants’ attitude.

## Methods

### Study design and population

The VGO study population originates from participants of a cross-sectional questionnaire survey (n = 14,163) among randomly selected GP patients (18–70 years old) living in small towns or villages in a livestock-dense area in the south of the Netherlands.^18^ Respondents who were willing to participate in a follow-up study and who were not working or living on a farm were eligible for a medical examination (n = 8,714). Based on their home addresses, 12 temporary research centers were established. Between March 2014 and February 2015, all respondents living within 10 km of one of these temporary research centers (n = 7,180) were invited to the nearest center for medical examination and 2,494 participated (response, 34.7%). The medical examination consisted among others of a second and more extended questionnaire and spirometry.^17,25^ The study protocol (13/533) was approved by the Medical Ethical Committee of the University Medical Centre Utrecht. All 2,494 subjects signed informed consent. In total, data from 37 subjects were excluded from the analyses because of missing data, resulting in a study population of 2,457 subjects.

### Medical examination

The questionnaire comprised among others items on education, profession, residential history, smoking habits, and respiratory health. Moreover, the questionnaire also contained 15 statements on attitude toward farming in their residential environment (statements are shown in Table 1). Statements were mostly adopted from a survey among the general Dutch population which was focused on the public’s view on intensive livestock farming.^16^ To assess lung function, pre- and postbronchodilator spirometry was conducted among 2,037 participants.^25^ We had two sources of information on pneumonia: (1) self-reported, physician-diagnosed pneumonia over the past 3 years, or (2) having had at least one pneumonia episode recorded in the EMR during the 3 years preceding the medical examination. Although our original finding was based on a combination of both sources,^22^ or EMR data alone,^24^ in the current analysis, we focused on the effect of attitude on associations with self-reported pneumonia because the impact of attitude was expected to be most pronounced for a self-reported outcome.

**Table 1:**
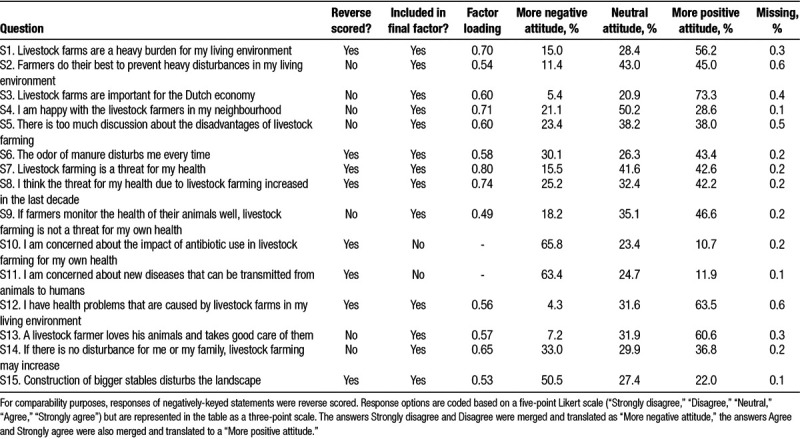
Statements regarding attitude toward farming in the residential environment and the distribution of 2,457 participants’ responses to the 15 statements

### Construction of a score for attitude toward livestock farming in the residential environment

Based on the 15 statements on attitude toward farming, we developed an “attitude-score” using factor analysis. Response options of the 15 statements were coded based on a five-point Likert scale (Table 1). Principal factor analysis was used to identify one or more latent factors that can be interpreted as an attitude toward farming. Standardized factor scores (z-scores, hereafter named attitude-score) were computed as linear combinations of scoring coefficients and standardized questionnaire responses for each participant, where a higher score indicates a more positive attitude toward farming.

### Livestock farm exposure variables

Distances between home addresses and livestock farms were computed using a geographic information system (ArcGis 10.1; Esri, Redlands, CA) as described previously.^18,25,26^ The following livestock farm exposure proxies were studied for each subject: (1) number of farms within 500 and 1,000 m, and (2) presence of a farm (pig, poultry, cattle, goat, sheep, horse) within 1,000 m (Yes/No).

### Data analysis

First, we assessed the association between the attitude-score and potential determinants using linear regression analysis. Results were expressed as regression coefficients (β) and 95% CIs representing the mean change in the attitude-score given a change in the determinant (one unit or otherwise stated in the Tables). The potential determinants of attitude studied were as follows: (1) personal characteristics, (2) respiratory health, and (3) exposure to livestock farms. Two adjusted models were assessed: model A, adjusted for age and gender, and model B, adjusted for age, gender, born in study area, childhood on a farm, BMI ≥ 30, visited a farm last 12 months, and high education.

Second, to study the impact of attitude on information bias (i.e., differential misclassification of self-reported pneumonia), we compared self-reported and EMR-based pneumonia, and computed sensitivity and specificity in a group with a more negative (< median attitude-score) and a more positive attitude (> median attitude-score). To study effect modification by attitude, the association between proximity to goat farms and pneumonia was also analyzed in the “more negative” and “more positive” group, and we tested interaction between farm proximity and attitude-score.

Third, sensitivity analyses were conducted after excluding subjects who attributed their symptoms to presence of livestock farms in their environment. The association between pneumonia and goat farm proximity (within 1,000 m as in Ref. ^22^) was analyzed with logistic regression, and expressed as odds ratios (ORs) and 95% CI. Data were analyzed using SAS 9.4 (SAS Institute Inc., Cary, NC).

More details on the study methodology are provided in the online supplement; http://links.lww.com/EE/A34.

## Results

### Study population

Participants were on average 56.4 ± 11.1 years old, and 54.6% of the study population consisted of women (Table 2). In total, 76.1% was born in the study area and one third (33.8%) had grown up on a farm. The number of missing answers to the 15 statements was low for all items (<0.6%) (Table 1). The majority of participants answered neutral or positive to all statements, with the exception of three statements regarding concerns about antibiotic usage in livestock farming, zoonotic diseases, and disturbance of the landscape due to construction of bigger sheds.

**Table 2:**
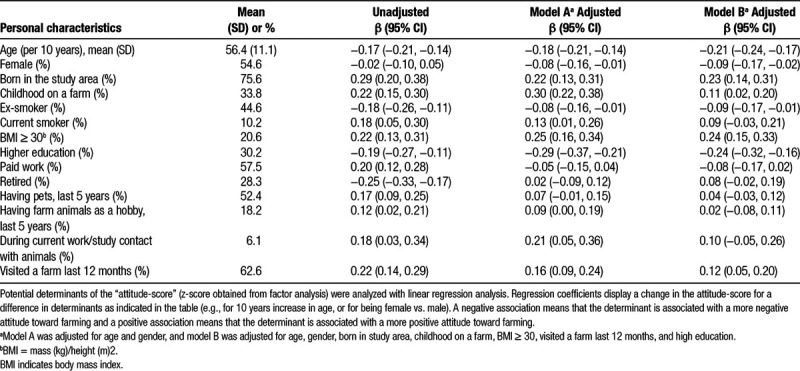
Characteristics of the study population of 2,457 adults from a general, nonfarming population, and association between potential determinants and the attitude-score

### Construction of attitude-score

After first exploratory factor analyses, statements 10 and 11 were removed because their residual correlation coefficients were >0.1. The final factor analysis was performed on the remaining 13 statements, and one latent factor was identified (eigenvalue = 5.14) and explained 97.6% of the total variance. Cronbach’s alpha was 0.89, suggesting a good internal consistency. Factor loadings (i.e., the correlations of the individual questionnaire items with the factor) ranged from 0.49 to 0.80 (Table 1). Including one of the initially removed statements (10 or 11) resulted in a very similar factor solution (correlation between factor scores based on 13 or 14 statements was 0.998).

### Determinants of attitude

Older participants, women, ex-smokers (vs. never smokers), and individuals with a higher education (vs. low and middle education) had a more negative attitude toward farming (Table 2). As expected, determinants related to familiarity with a farming environment—such as childhood on a farm, born in the study area, or a recent farm visit—were associated with a more positive attitude toward farming.

All self-reported respiratory health outcomes were associated with a lower attitude-score, whereas objectively measured respiratory health such as lung function and chronic obstructive pulmonary disease (COPD; based on lung function) was not associated with attitude (Table 3).

**Table 3:**
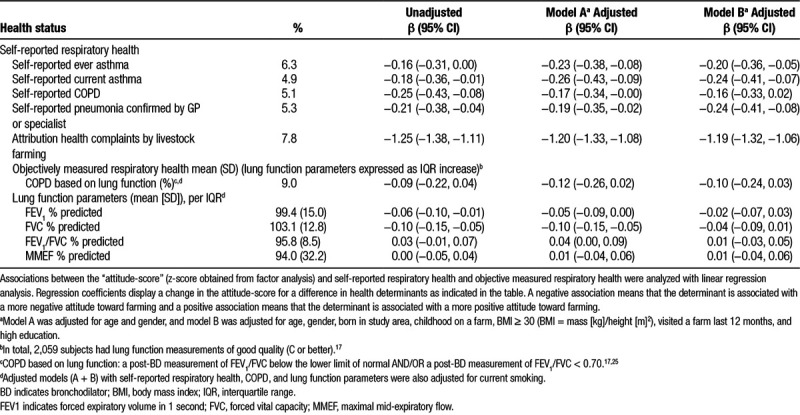
Associations between the attitude-score and self-reported and objectively measured respiratory health outcomes

The following proxy measures of livestock farm exposure were associated with a more negative attitude-score: a larger number of farms within 500 and 1,000 m of the home, presence of a pig farm (β, −0.13 [95% CI = −0.22, −0.04]), or a goat farm (β, −0.19 [95% CI = −0.31, −0.08]) within 1,000 m (supplementary Table S1; http://links.lww.com/EE/A34).

As expected, subjects who attributed their health complaints to livestock farming had a more negative attitude toward farming (Table 3). Excluding subjects who attributed their health symptoms to livestock farms in their environment (n = 191, 7.8%) did not change associations between attitude and personal characteristics and associations with farm exposures (data not shown). However, associations between the attitude-score and self-reported respiratory health symptoms were attenuated in the sensitivity analyses (data not shown).

### Impact of attitude on the association between proximity to goat farms and pneumonia

Sensitivity and specificity of self-reported pneumonia (compared with an EMR-based diagnosis) did hardly differ between those with a more negative attitude (sensitivity, 52%; specificity, 97%) and those with a more positive attitude (sensitivity, 56%; specificity, 98%). Residents living within 1,000 m of a goat farm had a higher risk of self-reported pneumonia (OR, 1.78 [95% CI = 1.07, 2.95]) (Figure 1), which differed slightly from the previously reported OR (2.0 [95% CI = 1.3, 3.1]) that was based on both EMR and self-reported pneumonia,^22^ and from the OR based on EMR only (2.3 [95% CI = 1.4, 3.9]).

**Figure.**
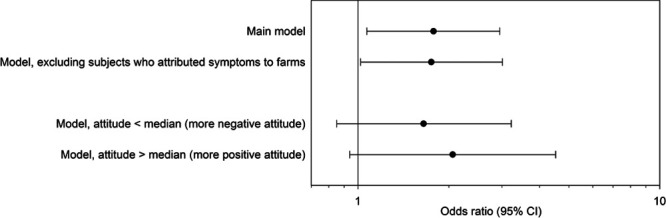
Effect of attitude on the previously observed association between self-reported pneumonia and living within 1,000 m of a goat farm (“main model”^22^).

No significant interaction was observed between attitude and living within 1,000 m of a goat farm (*P* value for interaction 0.63), suggesting that the association between goat farms and pneumonia was not modulated by attitude. In addition, dividing the population in a group with a more negative and a more positive attitude did not substantially change the association, but CIs were wider (< median attitude-score: OR, 1.65 [95% CI = 0.85, 3.23]; > median attitude-score: OR, 2.06 [95% CI = 0.94, 4.52]).

Excluding subjects who attributed their health symptoms to livestock farms in their environment did not change the association between self-reported pneumonia and living within 1,000 m of a goat farm (OR, 1.75 [95% CI = 1.02, 3.01]).

## Discussion

Our present study shows that the earlier observed association^22^ between proximity to goat farms and pneumonia in the Livestock Farming and Neighbouring Residents’ Health Study was not substantially biased by participants’ attitude toward farming.

Misclassification of self-reported pneumonia resulted in attenuated risk estimates when compared with EMR-based diagnosis, but misclassification was nondifferential with regard to participants’ attitude. Furthermore, the association between goat farm proximity and pneumonia was similar in groups with a more positive or more negative attitude (i.e., no effect modification), and excluding participants who attributed their health problems to livestock farming (7.8% of the population) did not meaningfully change the association. The attitude-score as defined in this article was used as a measure of information quality (quality of self-reported physical health). Because attitude is not a causal ancestor of physical health, it does not meet the causal structure required of a confounder or a causal intermediate. Adding the attitude-score as if it were a confounder hardly changed the association between goat farm proximity and self-reported pneumonia (OR, 1.73 [95% CI = 1.03, 2.93]).

We found several determinants that are associated with attitude toward farming in residential environments. In general, the study population had a relatively positive attitude toward farming. Most questions were answered with a neutral to positive tendency. Familiarity with farming could possibly explain the predominantly positive attitude. One third of the study population had grown up on a farm. The study area, in which 75.6% of the study population was born, is characterized by the highest farm density of the Netherlands. Previous studies on risk perception show that common risks are judged more acceptable than uncommon and unknown risks.^27^ Agricultural activities are familiar and common among the majority of the study population and therefore probably more acceptable. Attitude was indeed positively associated with determinants related to familiarity with a farming environment such as childhood on a farm, being born in the study area, or a recent farm visit.

We found that self-reported health symptoms were associated with a more negative attitude. Subjects who reported to attribute their health complaints to livestock farming had a much lower average attitude-score than other participants. This is in line with previous studies that showed positive associations between concern and reporting factors related to illness.^1,6^ Awareness bias^1^ might have played a role since we only observed an association between attitude and self-reported respiratory health and not with objectively measured respiratory health. Several indicators of livestock farm exposure were associated with a more negative attitude. Subjects who live in areas with a high number of livestock farms, especially in close proximity of pig and goat farms, had a more negative attitude toward farming than subjects living in areas with less livestock farms. The association with goat farms might be explained by an unprecedented outbreak of Q-fever, a zoonosis caused by *Coxiella burnetii*, that occurred in the study area between 2007 and 2010.^28^ Dairy goat farms with *C. burnetii*–induced abortions were implicated as the major source of infection in the neighboring human population. More than 3,500 acute Q-fever patients, mostly presenting as pneumonia, were officially registered, and it was estimated that 95 patients died. A study focused on regional differences in public perceptions regarding Q-fever found that this epidemic caused increased perceived anxiety and preventive behavior among subjects living in regions with high Q-fever incidence.^29^

The observed association with pig farms could possibly be explained by odor annoyance. Pig farms emit more offensive odor in comparison with cattle and poultry farms.^30^ Odor annoyance is common in populations living in the proximity of livestock farms and is a main source of annoyance.^31,32^ A Dutch study showed that the number of pigs, but also the number of poultry and cattle, around homes of residents was associated with odor annoyance.^33^

In 2011, a survey on the general public’s view of the Dutch population on intensive livestock farming was conducted.^16^ This survey consisted of two parts: a qualitative part that explored arguments that play a role in the discussion on intensive livestock farming in the Netherlands, and a second part that consisted of an online survey among 1,090 subjects from the Dutch general population. The 15 statements in our questionnaire were adopted from or inspired by this survey. Results of the online survey showed a lot of similarities with the answers to the statements given by our study population, even though our study population is living in a rural area with high livestock farm density. This might explain why our study population considers the benefits for the local (and Dutch) economy more important than the general Dutch population from the previous survey (73.3% vs. 52%). In the online survey, one of the most important arguments against intensive livestock farming was focused on potential risks for public health, and especially on antibiotic resistant bacteria and zoonotic diseases.^16^ The majority of our study population mentioned to be concerned about antibiotic usage in livestock farming and zoonotic diseases. The use of antibiotics in livestock production can lead to increased occurrence of antimicrobial resistance in bacteria which may transmit to humans.^34^ Previous studies show increased risks of livestock-related antimicrobial resistance among farmers with direct animal contact.^35,36^ This may have contributed to concerns about antimicrobial resistance in the study population, despite the large reduction of antimicrobial use of more than 60% in livestock farming since 2009 in the Netherlands.^37^ In the current VGO study, no increased risk was observed between farm proximity and carriage of extended-spectrum β-lactamase (ESBL)- and plasmid-encoded AmpC-producing Enterobacteriaceae.^19^ However, a slightly increased risk was observed between living near farms and carriage of livestock-associated methicillin-resistant *Staphylococcus aureus* (LA-MRSA), although the prevalence was low (0.4%), and there is a high likelihood of a chance finding.^20^ The Q-fever outbreak in the study area between 2007 and 2010 is likely to have contributed to our study population’s concerns on emerging zoonotic infections.^28,29,38^

Strengths of our study are our large, population-based sample and the low amount of missing data on the attitude statements. Both self-reported and objectively assessed data on respiratory health were available; this enabled us to compare associations with attitude and to explore awareness bias. Nevertheless, a number of limitations should be considered. First, the cross-sectional design makes it difficult to infer causality. Second, attitude toward farming may have contributed to the decision whether or not to participate to the medical examination and to the questionnaire survey where the study population originates from. Our previous studies showed that participants of the medical examination^17^ and responders to the questionnaire survey^18^ lived in closer proximity to farms compared with subjects who did not participate and with nonresponders, respectively. We have no information on attitude toward farming from the source population; therefore, it was not possible to analyze the effect of participation bias on the average reported attitude.

In conclusion, we developed an attitude-score to measure attitude toward farming in the residential environment. In general, the study population had a positive attitude toward farming, in particular if participants were more familiar with farming. Older participants, females, ex-smokers, and individuals with a higher education had a more negative attitude. Self-reported symptoms were also associated with a more negative attitude. However, we did not find any indication that the previously reported association between proximity to goat farms and self-reported pneumonia was biased by attitude. Overall, results of the current study indicate that attitude might play a role when using self-reported data in environmental health studies. When relying on self-reported data, we recommend to estimate attitude toward a potential hazard to assess the potential influence of awareness bias on epidemiologic associations.

## Conflicts of interest statement

The authors declare that they have no conflicts of interest with regard to the content of this report.

This work was supported by grant from the Lung Foundation Netherlands (grant number: 3.2.11.022) and funded by the Ministry of Health, Welfare and Sports and the Ministry of Economic Affairs of the Netherlands.

## Acknowledgments

The VGO study is conducted by a consortium (VGO consortium) of different research institutes (in alphabetic order): Institute for Risk Assessment Sciences of the Utrecht University (IRAS), Netherlands Institute for Health Services Research (NIVEL), National Institute for Public Health and the Environment (RIVM), Wageningen Bioveterinary Research of Wageningen University and Research (WUR), and Wageningen Livestock Research (WUR).

## Supplementary Material

## References

[1] Moffatt S Mulloli TP Bhopal R Foy C Phillimore P. An exploration of awareness bias in two environmental epidemiology studies. Epidemiology 2000 11 199 208 11021620 10.1097/00001648-200003000-00020

[2] Marcon A Nguyen G Rava M Braggion M Grassi M Zanolin ME. A score for measuring health risk perception in environmental surveys. Sci Total Environ 2015 527–528 270 278 10.1016/j.scitotenv.2015.04.110 25965040

[3] Coughlin SS. Recall bias in epidemiologic studies. J Clin Epidemiol 1990 43 87 91 2319285 10.1016/0895-4356(90)90060-3

[4] Baliatsas C Van Kamp I Bolte J Schipper M Yzermans J Lebret E. Non-specific physical symptoms and electromagnetic field exposure in the general population: can we get more specific? A systematic review. Environ Int 2012 41 15 28 22245541 10.1016/j.envint.2011.12.002

[5] Porsius JT Claassen L Smid T Woudenberg F Petrie KJ Timmermans DR. Symptom reporting after the introduction of a new high-voltage power line: a prospective field study. Environ Res 2015 138 112 117 25704831 10.1016/j.envres.2015.02.009

[6] Shusterman D Lipscomb J Neutra R Satin K. Symptom prevalence and odor-worry interaction near hazardous waste sites. Environ Health Perspect 1991 94 25 30 1954935 10.1289/ehp.94-1567940 PMC1567940

[7] Baliatsas C Bolte J Yzermans J. Actual and perceived exposure to electromagnetic fields and non-specific physical symptoms: an epidemiological study based on self-reported data and electronic medical records. Int J Hyg Environ Health 2015 218 331 344 25704188 10.1016/j.ijheh.2015.02.001

[8] Witthöft M Rubin GJ. Are media warnings about the adverse health effects of modern life self-fulfilling? An experimental study on idiopathic environmental intolerance attributed to electromagnetic fields (IEI-EMF). J Psychosom Res 2013 74 206 212 23438710 10.1016/j.jpsychores.2012.12.002

[9] Jacobs J Taylor M Agho K Stevens G Barr M Raphael B. Factors associated with increased risk perception of pandemic influenza in Australia. Influenza Res Treat 2010 2010 947906 23074650 10.1155/2010/947906 PMC3447299

[10] van Cleef BA Verkade EJ Wulf MW. Prevalence of livestock-associated MRSA in communities with high pig-densities in The Netherlands. PLoS One 2010 5 e9385 20195538 10.1371/journal.pone.0009385 PMC2828479

[11] Smit LA van der Sman-de Beer F Opstal-van Winden AW. Q fever and pneumonia in an area with a high livestock density: a large population-based study. PLoS One 2012 7 e38843 22685612 10.1371/journal.pone.0038843 PMC3369851

[12] Poulsen MN Pollak J Sills DL Casey JA Nachman KE Cosgrove SE. High-density poultry operations and community-acquired pneumonia in Pennsylvania. Environ Epidemiol 2018 2 e013 10.1016/j.ijheh.2017.12.005 PMC5880295 29268955

[13] Huijbers PM de Kraker M Graat EA. Prevalence of extended-spectrum β-lactamase-producing Enterobacteriaceae in humans living in municipalities with high and low broiler density. Clin Microbiol Infect 2013 19 E256 E259 23397953 10.1111/1469-0691.12150

[14] O’Connor AM Auvermann BW Dzikamunhenga RS. Updated systematic review: associations between proximity to animal feeding operations and health of individuals in nearby communities. Syst Rev 2017 6 86 28420442 10.1186/s13643-017-0465-z PMC5395850

[15] Statistics Netherlands Agriculture; crops, livestock and land use by general farm type, region.. http://statline.cbs.nl/Statweb.

[16] Verhue D Vieira V Koenen B van Kalmthout R. Opvattingen over megastallen [Views on intensive livestock farming]. Available at: http://edepot.wur.nl/168111. Accessed 14 August 2018

[17] Borlée F Yzermans CJ Aalders B. Air pollution from livestock farms is associated with airway obstruction in neighboring residents. Am J Respir Crit Care Med 2017 196 1152 1161 28489427 10.1164/rccm.201701-0021OC

[18] Borlée F Yzermans CJ van Dijk CE Heederik D Smit LA. Increased respiratory symptoms in COPD patients living in the vicinity of livestock farms. Eur Respir J 2015 46 1605 1614 26250492 10.1183/13993003.00265-2015

[19] Wielders CCH van Hoek AHAM Hengeveld PD. Extended-spectrum β-lactamase- and pAmpC-producing Enterobacteriaceae among the general population in a livestock-dense area. Clin Microbiol Infect 2017 23 120.e1 120.e8 27773759 10.1016/j.cmi.2016.10.013

[20] Zomer TP Wielders CC Veenman C. MRSA in persons not living or working on a farm in a livestock-dense area: prevalence and risk factors. J Antimicrob Chemother 2017 72 893 899 27999031 10.1093/jac/dkw483

[21] van Gageldonk-Lafeber AB van der Hoek W Borlée F. Hepatitis E virus seroprevalence among the general population in a livestock-dense area in the Netherlands: a cross-sectional population-based serological survey. BMC Infect Dis 2017 17 21 28056844 10.1186/s12879-016-2160-4 PMC5217153

[22] Freidl GS Spruijt IT Borlée F. Livestock-associated risk factors for pneumonia in an area of intensive animal farming in the Netherlands. PLoS One 2017 12 e0174796 28362816 10.1371/journal.pone.0174796 PMC5376295

[23] van Dijk CE Garcia-Aymerich J Carsin AE. Risk of exacerbations in COPD and asthma patients living in the neighbourhood of livestock farms: observational study using longitudinal data. Int J Hyg Environ Health 2016 219 278 287 26831047 10.1016/j.ijheh.2016.01.002

[24] Kalkowska DA Boender GJ Smit LAM. Associations between pneumonia and residential distance to livestock farms over a five-year period in a large population-based study. PLoS One 2018 13 e0200813 30016348 10.1371/journal.pone.0200813 PMC6049940

[25] Borlée F Yzermans CJ Krop E. Spirometry, questionnaire and electronic medical record based COPD in a population survey: comparing prevalence, level of agreement and associations with potential risk factors. PLoS One 2017 12 e0171494 28273094 10.1371/journal.pone.0171494 PMC5342260

[26] Smit LA Hooiveld M van der Sman-de Beer F. Air pollution from livestock farms, and asthma, allergic rhinitis and COPD among neighbouring residents. Occup Environ Med 2014 71 134 140 24142990 10.1136/oemed-2013-101485

[27] Slovic P. Perception of risk. Science 1987 236 280 285 3563507 10.1126/science.3563507

[28] van der Hoek W Morroy G Renders NH. Epidemic Q fever in humans in the Netherlands. Adv Exp Med Biol 2012 984 329 364 22711640 10.1007/978-94-007-4315-1_17

[29] Bults M Beaujean D Wijkmans C Richardus JH Voeten H. Q fever in the Netherlands: public perceptions and behavioral responses in three different epidemiological regions: a follow-up study. BMC Public Health 2014 14 263 24645896 10.1186/1471-2458-14-263 PMC4108011

[30] Ni JQ Robarge WP Xiao C Heber AJ. Volatile organic compounds at swine facilities: a critical review. Chemosphere 2012 89 769 788 22682363 10.1016/j.chemosphere.2012.04.061

[31] Wing S Horton RA Marshall SW. Air pollution and odor in communities near industrial swine operations. Environ Health Perspect 2008 116 1362 1368 18941579 10.1289/ehp.11250 PMC2569096

[32] Radon K Schulze A Ehrenstein V van Strien RT Praml G Nowak D. Environmental exposure to confined animal feeding operations and respiratory health of neighboring residents. Epidemiology 2007 18 300 308 17435437 10.1097/01.ede.0000259966.62137.84

[33] Hooiveld M van Dijk C van der Sman-de Beer F. Odour annoyance in the neighbourhood of livestock farming - perceived health and health care seeking behaviour. Ann Agric Environ Med 2015 22 55 61 25780829 10.5604/12321966.1141369

[34] Marshall BM Levy SB. Food animals and antimicrobials: impacts on human health. Clin Microbiol Rev 2011 24 718 733 21976606 10.1128/CMR.00002-11 PMC3194830

[35] Huijbers PM Graat EA Haenen AP. Extended-spectrum and AmpC β-lactamase-producing *Escherichia coli* in broilers and people living and/or working on broiler farms: prevalence, risk factors and molecular characteristics. J Antimicrob Chemother 2014 69 2669 2675 24879667 10.1093/jac/dku178

[36] Van den Broek IV Van Cleef BA Haenen A. Methicillin-resistant *Staphylococcus aureus* in people living and working in pig farms. Epidemiol Infect 2009 137 700 708 18947444 10.1017/S0950268808001507

[37] Veldman KT Mevius DJ Wit B van Pelt W Heederik D. MARAN 2018 Monitoring of Antimicrobial Resistance and Antibiotic Usage in Animals in the Netherlands in 2017 2018 Lelystad, The Netherlands Wageningen Bioveterinary Research (WBVR)

[38] Pijnacker R Reimerink J Smit LAM. Remarkable spatial variation in the seroprevalence of *Coxiella burnetii* after a large Q fever epidemic. BMC Infect Dis 2017 17 725 29157226 10.1186/s12879-017-2813-y PMC5697089

