# Supplementary material for: Attitude toward livestock farming does not influence the earlier observed association between proximity to goat farms and self-reported pneumonia
Source: Environ Epidemiol. 2019 Apr 12;3(2):e041. doi: 10.1097/EE9.0000000000000041 (PMC7952105; doi:10.1097/EE9.0000000000000041)
Supplement: Supplementary file 1 [file ee9-3-e041-s001.docx]

**Attitude towards livestock farming does not influence the earlier observed association between proximity to goat farms and self-reported pneumonia**

Floor Borlée, C. Joris Yzermans, Floor S.M. Oostwegel , François Schellevis, [VGO consortium], Dick Heederik, Lidwien A.M. Smit

**Supplementary Methods**

*Study design and population*

The VGO study was conducted in the eastern part of the province of Noord-Brabant and the northern part of Limburg, an area in the South of the Netherlands which is characterized by a high density of livestock farms. The study population originates from participants of a questionnaire survey (n=14,163) conducted in December 2012, as previously described by Borlée et al.^1^ Questionnaire respondents who were willing to participate in a follow-up study, and who were not working or living on a farm were eligible for a medical examination (n=8,714). Between March 2014 and February 2015, 7,180 persons were invited for medical examination and 2,494 participated (response 34.7%). The medical examination consisted amongst others of a second and more extended questionnaire and spirometry. More details about the recruitment of the study population and the medical examination have been described previously by Borlée et al.^2,3^

The study protocol (13/533) was approved by the Medical Ethical Committee of the University Medical Centre Utrecht. All 2,494 subjects signed informed consent. Patients’ privacy was ensured by keeping medical information and address records separated at all times by using a Trusted Third Party.

*Data collection*

*Questionnaire*

The questionnaire completed by the participants during the medical examination comprised amongst others items on education, profession, residential history, smoking habits, non-specific symptoms and diseases. Moreover, the questionnaire also contained items on attitudes towards farming in their residential environment. Statements were mostly adopted from a survey among the general Dutch population which was focused on the public’s view on intensive livestock farming.^5^

*Construction of score for attitude towards livestock farming in the residential environment*

The questionnaire included 15 statements related to attitudes towards livestock farming in the residential environment (see table 1). Response options were coded based on a five-point Likert scale: “Strongly disagree“, “Disagree”, “Neutral” (neither agree nor disagree), “Agree”, and “Strongly agree”. Responses to negatively-keyed statements were reverse-scored (see table 1). Correlation between the statements was checked with a Pearson correlation matrix. Principal factor analysis was used to identify one or more latent factors which can be interpreted as an ‘attitude towards farming’. The number of factors determined was based on the Kaiser-Guttman criterion (retain factors having an eigenvalue greater than 1), and by observing the point where the scree plot bends. Final factor analysis was done after excluding items with residual correlations or partial correlations < 0.1. Standardized factor scores (z-scores, hereafter named ‘attitude score’) were computed as linear combinations of scoring coefficients and standardized questionnaire responses for each participant, where a higher score indicates a more positive attitude towards farming. In total, data from 37 subjects were excluded from the analyses since they had more than 3 missing answers among the 15 statements (n=36), or answered all 15 statements with ”Strongly disagree” before recoding (n=1). Missing answers of all 2,457 remaining subjects were assigned to ”Neutral”.

*Spirometry*

Pre- and post-bronchodilator spirometry was conducted according to European Respiratory Society (ERS) guidelines and the European Community Respiratory Health Survey III (ECRHS-III),^6^ and is described in more detail by Borlée et al.^3^

*Livestock farm exposure variables*

Livestock farm exposure proxies were computed for each subject as described previously.^7^ In short, livestock farm proximity to the home address for each participant was determined using a geographic information system (ArcGis 10.1; Esri, Redlands, CA, USA). The following livestock farm exposure proxies were studied for each subject: 1) number of farms within 500 and 1000 m and 2) presence of a farm (pig, poultry, cattle, goat, sheep, horse) within 1000 m (Y/N).

*Data analysis*

First, we assessed the association between the attitude-score and potential determinants using linear regression analysis. Results were expressed as regression coefficients (β) and 95% confidence intervals (CI) representing the mean change in the attitude-score given a change in the determinant (one unit or otherwise stated in the Tables). The potential determinants of attitude studied were: 1) general characteristics: age, gender, born in study area, childhood on a farm, smoking habits, BMI, education, employment status, direct contact with (farm) animals; 2) health status: self-reported respiratory health, lung function (spirometry), 3) exposure to livestock farms. Potential confounders (general characteristics) with a p-value < 0.2 were selected beforehand by linear regression of attitude on potential confounders following a forward stepwise procedure based on improvement of Akaike’s Information Criterion (AIC). As a result, two adjusted models were run: the basic model A (only adjusted for age and gender) and the full Model B (age, gender, born in study area, childhood on a farm, BMI ≥ 30, visited a farm last 12 months, high education). Sensitivity analyses were conducted after excluding subjects who attributed their symptoms to presence of livestock farms in their environment.

Second, to study the impact of attitude on information bias (i.e. differential misclassification of self-reported pneumonia), we compared self-reported and EMR-based pneumonia, and computed sensitivity and specificity in a group with a more negative (< median attitude-score) and a more positive attitude (> median attitude-score). To study effect modification by attitude, the association between proximity to goat farms and pneumonia was also analysed in the ‘more negative’ and ‘more positive’ group, and we tested interaction between farm proximity and attitude-score. Freidl et al.^8^ found in the same study population (2,494 participants of the medical examination) a higher risk of pneumonia for residents living in close proximity to goat farms. Pneumonia was defined as self-reported physician-diagnosed pneumonia over the past three years, or pneumonia recorded in the Electronic Medical Records (EMR). In the current study, pneumonia was defined as only self-reported physician-diagnosed pneumonia over the past three years reported in the questionnaire because the impact of attitude was expected to be most pronounced for a self-reported outcome. The association between pneumonia and goat farm proximity (within 1,000 m as in^8^) was analysed with logistic regression, and expressed as odds ratios (OR) and 95% CI.

Third, a sensitivity analysis was conducted after excluding subjects who attributed their symptoms to presence of livestock farms in their environment.

Data were analysed using SAS 9.4 (SAS Institute Inc. Cary, NC, USA).

**References supplementary file**

1. Borlee F, Yzermans CJ, van Dijk CE, Heederik D, Smit LA. Increased respiratory symptoms in COPD patients living in the vicinity of livestock farms. *Eur Respir J*. 2015;46:1605-1614.
2. Borlee F, Yzermans CJ, Krop E, Aalders B, Rooijackers J, Zock JP, et al. Spirometry, questionnaire and electronic medical record based COPD in a population survey: Comparing prevalence, level of agreement and associations with potential risk factors. *PLoS One*. 2017;12:e0171494.
3. Borlee F, Yzermans CJ, Aalders B, Rooijackers J, Krop E, Maassen CBM, et al. Air Pollution from Livestock Farms is Associated with Airway Obstruction in Neighboring Residents. *Am J Respir Crit Care Med*. 2017;196:1152-1161.
4. Yzermans J, Baliatsas C, van Dulmen S, Van Kamp I. Assessing non-specific symptoms in epidemiological studies: Development and validation of the Symptoms and Perceptions (SaP) questionnaire. *Int J Hyg Environ Health*. 2016;219:53-65.
5. Verhue D, Vieira V, Koenen B, van Kalmthout R. Opvattingen over megastallen [Views on intensive livestock farming]. <http://edepot.wur.nl/168111>. Accessed August 14, 2018.
6. Burney P, Jarvis D. Lung function protocol, data sheets and lung function questionnaire. [www.ecrhs.org](http://www.ecrhs.org). Accessed August 14, 2018.
7. Smit LA, Hooiveld M, van der Sman-de Beer F, Opstal-van Winden AW, Beekhuizen J, Wouters IM, et al. Air pollution from livestock farms, and asthma, allergic rhinitis and COPD among neighbouring residents. *Occup Environ Med*. 2014;71:134-140.
8. Freidl GS, Spruijt IT, Borlee F, Smit LA, van Gageldonk-Lafeber AB, Heederik DJ, et al. Livestock-associated risk factors for pneumonia in an area of intensive animal farming in the Netherlands. *PLoS One*. 2017;12:e0174796.

**Supplementary table**

Table S1. Associations between the attitude-score and determinants of livestock farm exposure.

|  | **Mean (SD) or n (%)** | **Unadjusted**  **β (95%CI)** | **Model A^a^**  **Adjusted**  **β (95%CI)** | **Model B^a^**  **Adjusted**  **β (95%CI)** |
| --- | --- | --- | --- | --- |
| **Number of livestock farms, mean (SD)** | | | | |
| Nr of farms within 500 m | 1.8 (2.1) | −0.01 (−0.02, 0.01) | −0.01 (−0.03, 0.01) | −0.02 (−0.04, 0.00) |
| Nr of farms within 1000 m | 9.3 (5.9) | −0.01 (−0.01, 0.00) | −0.01 (−0.01, 0.00) | −0.01 (−0.02, −0.01) |
| Presence of farms within 1000 m per animal category, n (%) | | | | |
| Any farm | 2357 (95.9) | 0.09 (−0.10, 0.28) | 0.07 (−0.11, 0.26) | 0.02 (−0.17, 0.21) |
| Pig farm | 1949 (79.3) | −0.08 (−0.18, 0.01) | −0.09 (−0.18, 0.00) | −0.13 (−0.22, −0.04) |
| Poultry farm | 1356 (55.2) | 0.07 (0.00, 0.15) | 0.06 (−0.02, 0.13) | 0.01 (−0.07, 0.08) |
| Cattle farm | 2314 (94.2) | 0.05 (−0.11, 0.21) | 0.04 (−0.12, 0.20) | 0.01 (−0.15, 0.17) |
| Goat farm | 266 (10.8) | −0.12 (−0.25, 0.00) | −0.16 (−0.28, −0.04) | −0.19 (−0.31, −0.08) |
| Sheep farm | 771 (31.4) | −0.01 (−0.09, 0.07) | −0.02 (−0.10, 0.06) | −0.04 (−0.12, 0.04) |
| Horse farm | 1763 (71.8) | 0.04 (−0.05, 0.12) | 0.04 (−0.04, 0.12) | 0.02 (−0.06, 0.10) |

Associations between the ‘attitude-score ’ (z-score obtained from factor analysis) and determinants of livestock farm exposure were analyzed with linear regression analysis. Regression coefficients display a change in the attitude-score for a difference in determinants as indicated in the table. A negative association means that the determinant is associated with a more negative attitude towards farming and a positive association means that the determinant is associated with a more positive attitude towards farming.
^a^ Model A was adjusted for age and gender, model B was adjusted for: age, gender, born in study area, childhood on a farm, BMI ≥ 30 (BMI: body mass index = mass (kg)/ height (m)^2^), visited a farm last 12 months, high education
